# Evaluating the effect of diethyl ether and moringa oleifera antioxidant additives on the performance and emission characteristics of jatropha biodiesel-diesel blended fuel on CI engine – An experimental investigation

**DOI:** 10.1016/j.heliyon.2024.e31436

**Published:** 2024-05-20

**Authors:** Tewodros Taye Birhanu, Dinku Seyoum Zeleke

**Affiliations:** Department of Mechanical Engineering, Addis Ababa Science and Technology University, Addis Ababa, Ethiopia, 16417, Ethiopia

**Keywords:** Biodiesel production, Oxidative stability, Moringa antioxidant, Diethyl ether, Performance

## Abstract

Alternative fuels can be produced from both non-edible feedstocks and edible crops. The higher production costs and contaminating nature of vegetable biofuels, which cause engine component failure, make it conceivable to encourage the synthesis of biodiesel from non-edible sources. One of the most widely utilized alternative fuels is Jatropha biofuel, which has performance levels comparable to diesel fuels and can be used with CI (Compression Ignition) engines without any modifications. However when it comes to oxidative stability properties that impact shelf life and commercialization, the majority of biodiesels—including Jatropha—are lacking. Therefore, the objective of this study was to enhance the oxidative stability and other physicochemical parameters, such performance and emission characteristics, of Jatropha biodiesel with diesel blends by adding additives like DEE (diethyl ether) and MA (moringa oleifera antioxidant). The seeds of jatropha and moringa were harvested by hand and then mechanically extracted with a screw press. A conical flask containing the precisely weighed amount of oil is filled with 50 mL of neutral alcohol. The combination is then heated for an hour using a water condenser over a bath. Using phenolphthalein indicator, the contents are titrated with KOH solution after cooling. Weight of oil taken (w)/volume of KOH used (mL) × normality of KOH is the formula used to determine the acidity value of jatropha oil. It is therefore below the minimum level set by ASTM D 675, which is 2.5 mg KOH/g. Methanol was used in the transesterification process to produce biodiesel, and potassium hydroxide (KOH) was used as a catalyst. Then, using 5 % DEE and 10 % MA additives, the physicochemical properties of jatropha biodiesel—such as density, kinematics viscosity, calorific value, and oxidative stability—were characterized. The percentage of improvement of the biodiesel's mentioned properties with these additives was 0.68 %, 2.8 %, 0.73 %, and 33.8 %, respectively. The brake thermal efficiency (BTE) of B40MA10DEE05D45 increased by 8.52 % whereas the brake specific fuel consumption (BSFC) of B50MA10DEE05D35, which is Made up of 44 % diesel, 50 % jatropha biodiesel, 5 % DEE, and 10 % MA fuels, declined by 5.14 %. As a result of these additions, the blended fuel's CO, HC, and NOx emissions were reduced by 3.51 %, 2.25 %, and 8.64 %, respectively. Therefore, a 20 % blend of Jatropha biodiesel and diesel containing antioxidants from Moringa can be used in compression ignition engines without the need for engine modifications and with high oxidation stability.

## 1 Introduction

The rate at which energy sources are being consumed is rising faster as a result of the expanding modernization and industrialization trend in our globe [1,2]. Alternative energy sources are becoming more popular due to the growing demand for fossil fuels and their environmental effects [[3], [4], [5], [6]]. Biodiesels are among the various alternative energy sources that replace conventional diesel after the oils of their respective feedstocks are treated through esterification and/or *trans*-esterification to improve their physical and chemical properties. This is because utilising these oils directly as engine fuel causes a wide range of problems, including low atomization, high emissions from incomplete combustion, injector clogging, carbon buildup, and oil rings sticking because of the oil's increased viscosity [6,7]. Vegetable oils were previously utilized as alternative fuels by compression ignition (CI) engines because of their higher cetane number and calorific values [8].

However, a number of research on the performance, combustion, and emission characteristics of biodiesels Made from edible vegetable oils have been undertaken recently, and the results show that these are lower [9]. Because of the issues surrounding the use of vegetable oils as fuel, researchers are currently interested in the use of biofuels derived from inedible sources [10,11]. The problems of vegetables oils are associated to their higher viscosity that leads poor atomization and in turn infects the injectors. Poor atomization also causes accumulation of deposits in the cylinder due to the burning of residuals [11]. Another problem with vegetable oils is that the cost of production of biofuels from these sources accounts higher price and hence production of biofuels from non-edible sources is being preferable, and hence jatropha biodiesel is the most desirable one [5].

In addition to the substitution of crude diesel, biodiesels are excellent in their lubricating nature for the engine fuel system components. The lubricity character of the biodiesels reduces any fuel system component caused by excess friction in injectors and fuel pumps. Diesel is having the lower lubricity and higher wear scar and it needs additives to improve its lubricity. Hence biodiesels have excellent lubricity, they can also be used as lubricity improver additives. But the lubricity characteristics of biodiesels is also depending on the type of raw Materials in which the biodiesel is produced from. However, jatropha biodiesel is with the highest lubricity (95 %) and sunflower takes the least one with almost no lubricity. Therefore, blending jatropha biodiesel with diesel plays a vital role in improving the fuel system components lubricity also [12].

Jatropha oil can easily be extracted from its dry seeds through a number of mechanisms including mechanical and solvent extraction methods [13]. Method of oil extraction plays a significant role to achieve the optimum physicochemical properties of the oil and hence mechanical extraction method gives enhanced physicochemical properties of the oil when compared to hydraulic, solvent and Soxhlet extraction methods [5]. The moisture and free fatty acid (FFA) content of the extracted oils are the basic parameters that determine if the oil is expected to be esterified or not. If the FFA content of the oil is higher than 3 %, the oil should be esterified before transesterification reaction takes place. Esterification and *trans*-esterification processes are Mainly used to convert the FFA available in the oil to fatty acid methyl esters and triglycerides into biodiesels respectively [14].

Transesterification process is the most commonly used method of biodiesel production with highest degree of efficiency, technologically simple and economically feasible features [15]. During biodiesel production through this method, there are a number of parameters that should be considered and kept optimized to improve its yield and quality. The common factors include: alcohol to oil molar ratio, temperature of reaction, catalyst concentration and time of reaction [14,16,17]. Methanol is the most popular alcohol that has maximum biodiesel yield when compared to that of ethanol and butanol [14,17]. The physiochemical properties of the biodiesels which includes calorific value, density, viscosity and flash point are used to characterize its combustion performance and emission characteristics [5,18]. Specifically, jatropha biodiesel is poor in oxidative stability characteristics and adding a certain amount of antioxidant additives were found to be essential in order to improve its storage requirements [1]. In order to overcome such problems, a number of researchers have explored different types of organic compounds as additives and among these, nanoparticles are the most common ones.

Organic compounds like oxides of Manganese (MnO_2_), Cupper (CuO_2_), Magnesium (MgO2), Iron (Fe_2_O_3_) and Nickel (NiO_2_) are commonly used synthetic additives of diesel and biodiesels to improve some of their physicochemical properties. The use of these compounds May also improve engine performance and exhaust emissions. However, these synthetic additives are having adverse effects on the oxidative stability of the diesel and biodiesel fuels and engine components. Hence most of the fuel tanks of engines are Made up of these metals that the organic additives are Made and when added as additives over the fuels, they accelerate the rate of oxidative deterioration because of metal to metal reaction initiates the free radical formation and that cause oxidation to take place as well as the fuel tank and other fuel system components failure [19,20].

The oxygenated nature and antioxidant property of moringa oleifera biodiesel made it a potential substitute of diesel fuels. Moreover, the use of this biodiesel as additive for or as a dual blend with other biodiesels played a significant role in improving the oxidative stability of the blended fuels. The unsaturated free fatty acid content of moringa oil/biodiesel was lower, and hence it is having a best anti-oxidant characteristic [21,22]. On the other hand, ethers are not only used as alternative substitutes of conventional energy sources but can also be used as additives for these conventional and alternative fuels to improve their physicochemical properties, engine performance and emission characteristics [23,24]. The kinematics viscosity, flash point and cetane number of a biodiesel before DEE additive were 4.5 mm^2^/s, 165 °C and 51 while after the addition of 5 % by volume of DEE were found to be 4.2 mm^2^/s, 148 °C and 54 respectively [25]. The use of 5 % DEE additive for 10 % and 20 % jatropha biodiesel and diesel blend improved the brake specific fuel consumption and brake thermal efficiency with percentage decrement of BSFC 3.7 % and 3.5 % and with percentage increment 6.1 % and 6.2 % of BTE respectively [26]. The use of DEE was used to improve the emission characterization of the engine also. The NOx emission of biodiesels is known by its higher yield because it is directly dependent on oxygen concentration, mixture formation and cylinder flame temperature [23,25]. This Makes it more plentiful and easier to produce than conventional fossil fuels, which Makes it renewable and relatively less costly. 100 % by volume of soybean methyl ester is referred to as SME, and 100 % by volume of rapeseed methyl ester is known as RME. The heat release rate figure shows that the Blend 82's increased compression ratio led to a longer combustion period and ignition delay when compared to diesel fuel [[26], [27], [28]].

Therefore, the current study primarily examined the use of DEE (Diethyl Ether) and Moringa oleifera antioxidant (MA) additives to improve the physicochemical properties, engine performance, and emission characterization of jatropha biodiesel and its blends with diesel for a maximum engine speed of 3250 and an engine load ranging from 0 % to 100 %. Hence, there are currently not many published studies on the usage of DEE and MA additives on diesel blended fuel and jatropha in CI engines. However, given their increased physicochemical qualities, performance, and emission characteristics, the use of DEE and MA as additives to biodiesel diesel blended fuels will be the ideal research moving forward.

## 2 Materials and methodology

### 2.1 Jatropha and moringa seed oil production

The production of oil of both moringa and jatropha was carried out using screw press because of its lower cost, easy to operate, quick for production of oil and do not contaminate the oil because it does not use any chemicals for extraction. The total yield of the oil for jatropha and moringa was 4.95 L and 0.67 L out of from 18.6 kg jatropha and 2.3 kg moringa seed kernels and the percent of oil produced is found ≈24.3 % and ≈26.5 % respectively. Once the oils have been produced, the task of degumming and purifying of the oils was followed and the yield of the oil after degumming was 89.7 % for jatropha and 91.2 % for moringa oil. Even-though mechanical extraction method has several advantages, the yield of the oil was lower when compared to that of solvent extraction method [10,11]. The extraction efficiency of mechanical method was 39 % while that of chemical extraction method using hexane was found to be 98 % [11]. The task of degumming is a task of reducing the phospholipid content of the bio-oil, and improves the properties of the oil specifically viscosity and carbon residuals. Because of degumming, both of these properties were found to be decreased [29]. It is used to reduce the acid value of the oil by removing the gums that cause the yield of the biodiesel to be affected [30].

### 2.2 Jatropha and moringa biodiesel production

The process of biodiesel production of both jatropha and moringa was performed through transesterification process using alkali catalyst. Trans means cross and ester is the name of a specified type of chemical bond in the oil and jointly the meaning of transesterification is the process of crossing and making ester bonds from triglycerides to produce biodiesels. It was carried out using 500 ml capacity three naked flask with 1, 1.25 and 1.5 % catalyst concentration (KOH), 6, 9 and 12 methanol to oil molecular ratio, at a steering speed of the reaction 500 rpm and time of reaction was 1.5 h at a temperature of 65 °C as presnetd in Table 1.

During the production process, methanol was used as an alcohol because of its higher yield than ethanol and butanol for both jatropha and moringa oils [14]. Methanol was used as alcohol not only for its higher yield but also for improved physicochemical properties, performance and emission characterization of the biodiesel. For 1.5 % of KOH with 12:1 oil to methanol ratio shows highest yield of biodiesel which is 96.8 % of its oil. On the other hand, moringa biodiesel production was carried out according to the optimized biodiesel production using 1 % KOH catalyst and 6:1 methanol to oil ratio with 65 °C reaction temperature at 450 rpm for 1hr [31]. The yield was found to be 92.3 % which is lower when compared to that of jatropha biodiesel yield. Different blend ratios of diesel and jatropha biodiesel with and without 5 % DEE and 10 % MA additives were prepared after the biodiesel was produced, as presented in Table 2.

**Table 1:** Physicochemical properties of jatropha (JO) and moringa oils (MO) and their biodiesels.

No.	Physicochemical property				
		JO	MO	JME	MME
1	Average Moisture Content	4.92	4.127	0.021	0.019
2	Average acid value	1.796	1.58	0.43	0.41
3	Average saponification value	207.57	183.2	–	–
4	Average peroxide value	2.67	–	4.93	–

**Table 2:** Detail specifications of diethyl ether.

Data	Specifications
Manufacturer	Sigma Aldrich
Chemical Formula	(CH_3_)_2_O
Purity	>99.5 %
CAS No.	60-29-7
Molecular Weight	74.12
Auto ignition temperature	160 °C
Flash Point	−45 °C
Boiling Point	34.6 °C
Density	713 kg/m^3^
Kinematics Viscosity @ 25 °C	0.224 mm^2^/s
Cetane Number	125

### 2.3 Preparation of diesel-biodiesel blended fuel with DEE and MA additives

Diesel was purchased from locally recognized petrol station and DEE which is a product of Sigma Aldrich (99.7 % quality level, >99 % assay, 160 °C auto ignition temperature, in the form of liquid state) was used for the blended fuel as additive. Then, blends of jatropha biodiesel with and without 5 % DEE and 10 % MA additives were tested, with percentages of 10 %, 20 %, 30 %, 40 %, and 50 % over diesel fuel. The blended fuels without additives are denoted by the following nomenclature: B10MA00DEE00D90, B20MA00DEE00D80, B30MA00DEE00D70, B40MA00DEE00D60, B50MA00DEE00D50, and B100MA00DEE00D00; the blended fuels with additives are denoted by the following blends: B10MA10DEE05D75, B20MA10DEE05D65, B30MA10DEE05D55, B40MA10DEE05D45, and B50MA10DEE05D35, respectively, for the previously mentioned percentage of biodiesels over diesel. Table 3 provides detailed information about biodiesel-diesel blended fuel.

**Table 3:** Details description of diesel-biodiesel blended fuels with additives.

Blended Samples by Vol.	Blend Concentration												
	Diesel	B10MA00DEE00D90	B10MA10DEE05D75	B20MA00DEE00D80	B20MA10DEE05D65	B30MA00DEE00D70	B30MA10DEE05D55	B40MA00DEE00D60	B40MA10DEE05D455	B50MA00DEE00D50	B50MA10DEE05D35	B100MA00DEE00D00	B85MA10DEE05D00
JB (%)	0	10	8.5	20	17	30	25.5	40	34	50	42.5	100	85
MA (%)	0	0	1	0	2	0	3	0	4	0	5	0	10
DEE (%)	0	0	0.5	0	1	0	1.5	0	2	0	2.5	0	5
D (%)	100	90	90	80	80	70	70	60	60	50	50	0	0

Where; B = Jatropha Biodiesel, MA = Moringa Antioxidant, DEE = Diethyl Ether, D = Diesel.

Blend ratio Nomenclature = B00MA00DEE00D00.

### 2.4 Physicochemical properties characterization

In this research, the physicochemical properties like density, kinematics viscosity, calorific value, oxidative stability, flash point and cold flow properties of the jatropha biodiesel-diesel blended fuel with and without DEE and MA additives were determined. The lowest temperature at which fuel vapor exposed to an open flame can ignite is known as the flash point is very important physiochemical property. Likewise the energy content of the fuel is represented by the calorific value, sometimes referred to as the heating value. The calorific value of jatropha biodiesel is lower than that of petroleum diesel, which may affect engine performance. When compared to petroleum diesel, jatropha biodiesel is typically more prone to oxidation. Addition of moringa antioxidants is common method to enhance oxidative stability. In general, jatropha biodiesel has a higher kinematic viscosity than petroleum diesel. This characteristic has an impact on engine lubrication and fuel flow. However, it can be managed by adding additives [18].

**Fig. 1:**
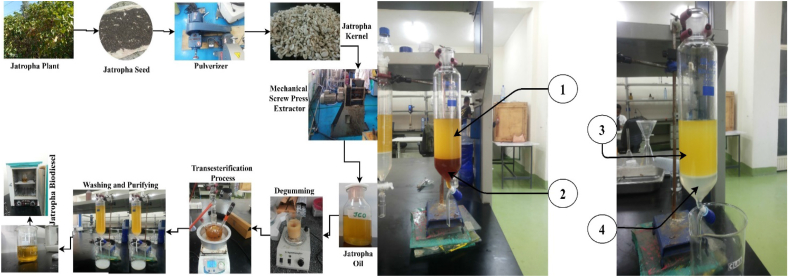
The experimental setup for the biodiesel production of jatropha and moringa 1) Biodiesel before washed, 2) Glycerol, 3) biodiesel after washed, 4) washing water.

The blended fuel density, viscosity, and calorific value, as well as their defined physicochemical qualities, are critical parameters that determine the engine's performance and emission characteristics. Therefore, Fig. 1 depicts the biodiesel extraction procedure. In contrast, stability and flash point are utilized to evaluate the biodiesel sample's handling and shelf life (commercialization) [27]. Table 4. Physicochemical properties of the blended fuel were demonstrated using standard test methods from ASTM and EN. As a result, Table 5 presents the physicochemical characteristics of jatropha biodiesel and its mixes. The results and discussion section will address the determined physiochemical parameters in detail. Therefore, when compared to biodiesel made from palm oil, biodiesel made from jatropha oil has a number of benefits, including superior cold flow characteristics, a lower pour point, cloud point, and cold filter plugging point. Jatropha biodiesel, however, has an oxidation stability of only 2.82 h, which makes it unacceptable for use in diesel engines. Hence, adding 10 % moringa antioxidant, as shown in Table 5, allows for the direct use of jatropha biodiesel in diesel engines without requiring engine modifications. Finally, adding 10 % moringa antioxidant extends the oxidation stability of jatropha biodiesel from 2.82 h to 15 h. Consequently, using jatropha biodiesel in a diesel engine has no impact on the engine.

**Table 4:** ASTM (American society for testing and materials) and EN (European).

No.	Physicochemical Properties	ASTM D6751		EN 14214	
		Method	Limit	Method	Limit
1	Calorific Value				
2	Kinematics Viscosity (40 °C)	D 445	1.9 - 6	ISO 3104	3.5–5
3	Density (15 °C)	D 1298	880	ISO 3675/12,185	860–900
4	Flash Point %C	D 93	130	ISO 2719	101
5	Cloud Point	D 2500	−12–−3	EN 23015	–
6	Pour Point	D 97	−16–−15		–
7	Acid Value	D 664	0.5	EN 14104	0.5
8	Oxidative Stability (hrs)		3	EN 14112	8

**Table 5:** Physicochemical properties of jatropha biodiesel and its Blends.

**No.**	**Blends (Samples)**	**ρ @ 15^C (kg/m**^**3**^**)**	**KV @ 40%C (m**^**2**^**/s)**	**CV MJ/kg**	**FP** **%C**	**FiP** **%C**	**CP** **%C**	**PP** **%C**	AV mg KOH/g	SV mg KOH/g	PV mEq/kg
**1**	**Diesel**	830	**3.40**	48.05	52.6	57.3	-11.3	-13.7			
**2**	**B10MA00DEE00D90**	835	3.45	47.52	58.9	63.8	-10.2	-12.5			
**3**	**B10MA10DEE05D75**	828	3.48	48.01	56.1	60.7	-10.9	-13.1			
**4**	**B20MA00DEE00D80**	842	3.60	46.61	64.8	71.3	-9.1	-11.9			
**5**	**B20MA10DEE05D65**	832	3.48	47.17	57.3	63.2	-10.1	-12.4			
**6**	**B30MA00DEE00D70**	845	3.62	45.73	65.3	72.9	-8.6	-10.8			
**7**	**B30MA10DEE05D55**	841	3.53	46.05	59.1	65.3	-9.3	-11.1			
**8**	**B40MA00DEE00D60**	852	3.75	45.18	69.7	79.4	-7.9	-10			
**9**	**B40MA10DEE05D45**	845	3.59	45.89	60.5	67	-8.5	-10.4			
**10**	**B50MA00DEE00D50**	855	3.88	44.29	73.2	92.4	-7.3	-9.6			
**11**	**B50MA10DEE05D35**	853	3.51	44.52	63.1	71.3	-7.6	-9.8			
**12**	**B1000MA00DEE00D00**	876	4.68	43.32	179.6	159.2	-6.1	-8.2	0.78	-	2.67
**13**	**B85MA10DEE05D00**	869	4.58	43.63	78.4	83.2	-6.5	-8.3	-	-	-
**14**	**JO**	913	3.40						1.796	207.6	-
**15**	**MO**	910							1.58	183.2	-
**16**	**MB**	873							0.71		4.93

Where; ρ = Density, KV = Kinematics Viscosity, CV = Calorific Value, FP= Flash Point, FiP = Fire Point, CP = Cloud Point, PP = Pour Point, AV = Acid Value, SV = Saponification Value, PV = Peroxide Value

### 2.5 Engine test experimental setup

**Fig. 2:**
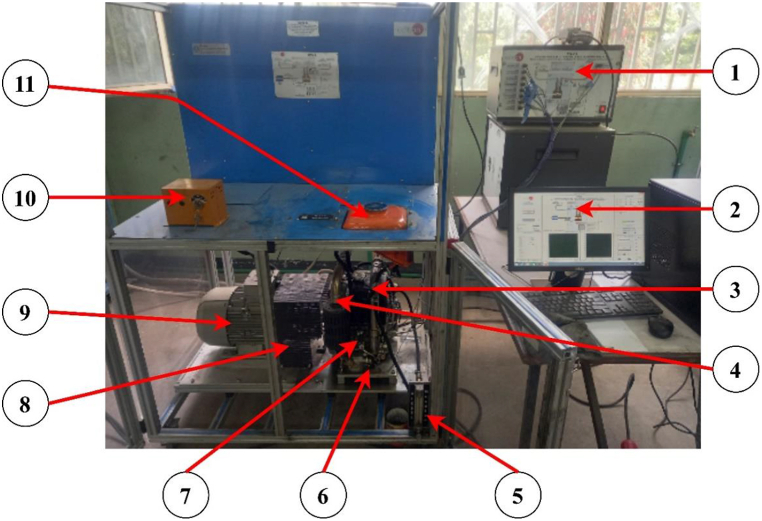
Experimental setup for TBMC8 single cylinder CI engine test bench 1) Control interface (SCADA), 2) Computer, 3) Intake MAnifold, 4) Exhaust MAnifold, 5) Fuel flow meter, 6) Throttle valve, 7) Diesel engine, 8) Coupling, 9) Dynamometer (Motor), 10) Ignition switch, 11) Fuel tank.

**Fig. 3:**
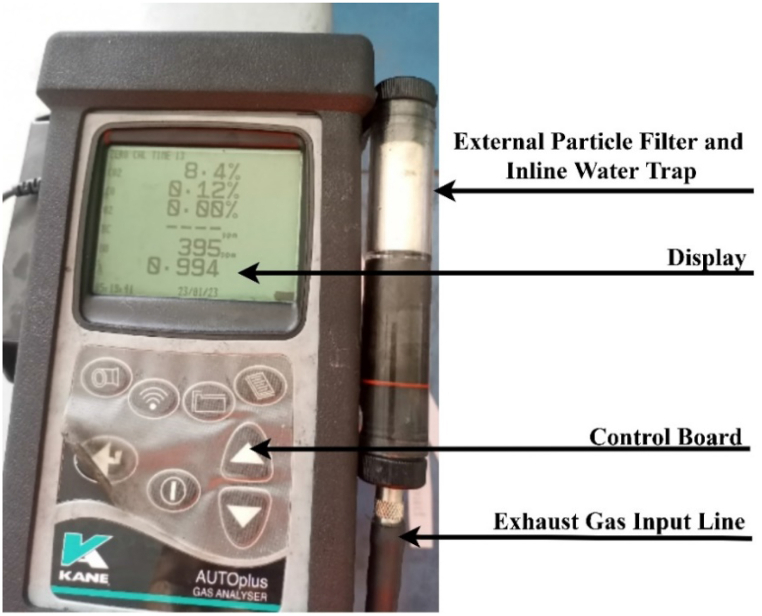
Kane AUTO-plus five gas analyzer.

The 7.5 kW single-cylinder, four-stroke computer-controlled TBMC8 test bench, depicted in Fig. 2, was utilized to assess the engine's performance. Likewise, as shown in Fig. 3, the exhaust emission was examined using a Kane AUTO-plus five gas analyzer. Exhaust emissions such as CO, CO2, HC, and NOx are examined along with engine performance metrics such as braking torque, brake power, brake thermal efficiency, and specific fuel consumption. A 400 ml sample from each mix was added to the gasoline tank of the engine, and it was driven for roughly 15 min. The SCADA software of the engine recorded the engine performance parameters. Following the completion of one test, the fuel was drained out, the next fuel blend was added to the fuel tank, and the engine was run for an additional 5 min in order to burn off any fuel residue that might still be in the fuel line. Also, pure diesel was utilized to wash off any remaining fuel residue in the fuel line in between testing batches of blended fuels. Table 6, Table 7 provide the test engine and exhaust emissions analyzer specifications, respectively.

**Table 6:** Specifications of TBMC8 single cylinder CI engine test bench.

No.	Engine Parameters	Engine Features
1	Engine Type	Single cylinder, four-stroke, water cooled, computer-controlled, diesel (CI) engine
2	Speed Range	0–6000 RPM
3	Power Range	0–7.5 KW
4	Fuel Flow Rate	2–40 ml/min
5	Inlet Air and Outlet Gases Flow Rate	0–400 m^3^/h
6	Pressure	0–1 Bar
7	Induction Type	Naturally aspired
8	Starting	Electrically (Motor assisted)

**Table 7:** Specifications of Kane five gas analyzer.

No.	Exhaust Gases	Units	Range of Emissions		Reading Resolution	Accuracy (%)
			Minimum	MAximum		
1	CO	%	0	10	0.01	±5
2	CO_2_	%	0	16	0.1	±5
3	HC	ppm	0	5000	1	±5
4	NO_x_	ppm	0	1500	1	±5

### 2.6 Engine performance and emission characterization

The experimental investigation in this research was conducted with pure diesel and different grades of jatropha biodiesel-diesel blended fuel with and without DEE and MA additives. The performance parameters like; brake power (BP), brake torque (BT), brake specific fuel consumption (BSFC), brake thermal efficiency (BSFC) and exhaust gas temperature (EGT) have been discussed. The engine test was carried out on B10MA00DEE00D90, B10MA10DEE05D75, B20MA00DEE00D80, B20MA10DEE05D65, B30MA00DEE00D70, B30MA10DEE05D55, B40MA00DEE00D60, B40MA10DEE05D45, B50MA00DEE00D50, and B50MA10DEE05D35. The above biodiesel blends are with 5 % DEE and 10 % MA additives. The test engine was run between 0 and 80 % loads at a parental increment of 10. DEE and MA additives for the biodiesel diesel blends in this research were selected because of their improved performance and oxidative stability characteristics [28].

### 2.7 Uncertainty analysis

Several variables might cause uncertainty in an experiment, including the type of instruments used, the measurement method, the environment, and the experimental setup. Engine performance and emissions were measured for every scenario after applying the engine loads and speed for 10 min to ensure the measured values stayed the same. The experiment was conducted three times for each fuel. The root mean square of the uncertainty in the experimental data and the instrumental uncertainty was used to quantify the total uncertainty computed with observed parameters such as brake torque, exhaust gas temperature, Carbon monoxide, Carbon dioxide, and Nitrogen oxides [24]. A specific function, F, is influenced by a number of independent variables, x1, x2, x3, and so on. Moreover, wF represents the total percentage error, and the independent variable errors are w1, w2, …, and wn. Using Taylor's theorem, an error analysis was carried out to guarantee the accuracy of the test results. For those variables, the total measurement uncertainty is 1.97 %, which is below the 5 % standard deviation limit. equation (1) used to calculate the overall level of uncertainty:

(1) $$Overall\;uncer\;\tan\;ity=(\sqrt{{\left(\Delta BP\right)}^{2}+{\left(\Delta BT\right)}^{2}+{\left(\Delta BTE\right)}^{2}+{\left(\Delta BSFC\right)}^{2}+{\left(\Delta EGT\right)}^{2}+{\left(\Delta CO\right)}^{2}+{\left(\Delta CO_{2}\right)}^{2}}$$

## 3 Result and discussion

### 3.1 Characterization of jatropha and moringa biodiesels

The characterization of the physicochemical properties of jatropha biodiesel, moringa biodiesel and the blends of jatropha and diesel with and without DEE and MA additives was carried out according to ASTM D6751 and EN 14,214 standard test methods and fulfilled the required parameters. Characterization of physicochemical properties like oxidative stability, density, kinematics viscosity, calorific value and flash point including cold flow properties were investigated on the blends B00MA00DEE00D100, B10MA00DEE00D90, B10MA10DEE05D75, B20MA00DEE00D80, B20MA10DEE05D65, B30MA00DEE00D70, B30MA10DEE05D55, B40MA00DEE00D60, B40MA10DEE05D45, B50MA00DEE00D50, B50MA10DEE05D35, B100MA00DEE00D00, and B85MA10DEE05D00. The physicochemical properties of the biodiesel-diesel blended fuels with and without DEE and MA additives are presented in Table 5. Therefore, by adding 10 % of moringa antioxidant, the oxidation stability of jatropha biodiesel increased from 2.82 h to 15 h. Significant improvement in oxidation stability is maintained with these additives, even at high blend ratios of B40MA10DEE05D45.

#### 3.1.1 Oxidative stability testing

Jatropha biodiesel has a poor oxidative stability. Antioxidant must therefore be added to the mixture in order to increase the oxidative stability of jatropha biodiesel. Moringa biodiesel, on the other hand, is an excellent antioxidant source when added to biodiesels like jatropha and others that have poor oxidative stability [32]. Moringa biodiesel added at 3 %, 5 %, and 10 % by volume to jatropha biodiesel was examined in this study using a professional Rancimat to determine its oxidative stability. With an induction period of 2.82 h, jatropha had low stability characteristics, while moringa exhibited the maximum stability characteristics, requiring a period of 20.69 h. However, jatropha biodiesel with additives of 3 %, 5 %, and 10 % moringa methyl ester was shown to have an oxidative stability of 3.93 h, 5.26 h, and 15 h, respectively. This indicates that, in accordance with EN 14112 standard, which has a minimum limit of 8 h, the addition of 10 % MA to the jatropha biodiesel demonstrated an excellent improvement in oxidative stability. Biodiesel that has a higher oxidative stability will oxidize more steadily and be a better fit for commercialization [33].

#### 3.1.2 Density

The density of the blended fuel with and without DEE and MA additives were measured using hydrometer and the result is depicted in Table 5. The densities of the blended fuels were increasing as the concentration of the biodiesel in the blend was increasing. The percentage increment of density for the blended fuels: B10MA00DEE00D90, B20MA00DEE00D80, B30MA00DEE00D70, B40MA00DEE00D60, B50MA00DEE00D50 and B100MA00DEE00D00 as compared to diesel was 0.6 %, 1.45 %, 1.81 %, 2.65 %, 3 % and 5.5 % respectively. The density of the mixed fuels, on the other hand, fell when additives were used, and the percentage decrease was 0.8 %, 1.19 %, 0.47 %, 0.82 %, 0.23 %, and 0.8 % with respect to the blended fuels that were previously indicated. As a result, DEE has a lower density; hence, adding it to the blended fuel significantly reduced its density. Similar results are investigated by L. S. de Sousa et al. [34].

#### 3.1.3 Kinematics viscosity

At a temperature of 40 °C, the kinematic viscosity (KV) of diesel and jatropha biodiesel blends with and without DEE and MA additives were determined using a Modular Compact Rheometer MCR 102 in accordance with ASTM D445; the results were 3.75m2/s and 3.59m2/s, respectively. The detail blends kinematic viscosities are presented in Table 5. When the blend's biodiesel concentration rose, viscosity increased for each mixture. For B10MA00DEE00D100, B20MA00DEE00D80, B30MA00DEE00D70, B40MA00DEE00D60, B50MA00DEE00D50, and B100MA00DEE00D00, the percentage increment of kinematic viscosity in relation to diesel was determined to be 1.47 %, 5.88 %, 6.47 %, 10.29 %, 14.12 %, and 37.65 %, respectively. The blend fuel's kinematic viscosity was, however, reduced by 2.26 %, 3.33 %, 2.49 %, 4.27 %, 9.54 %, and 2.14 % when 5 % DEE and 10 % MA additives on the blended fuel were used. Blends of jatropha biodiesel with diesel have reduced kinematic viscosity due to the lower kinematic viscosity of the DEE ingredient (0.233 cSt at 40 °C) [25,26].

#### 3.1.4 Calorific values

Adiabatic bomb calorimeter 1241 - EF was used to evaluate the calorific value of various grades of jatropha biodiesel with diesel blends with and without additives in accordance with ASTM D240 standard. In contrast, the addition of 5 % DEE v/v and 10 % MA v/v additives raised the calorific value of the corresponding blend. Table 5 illustrates how the calorific values of diesel and jatropha biodiesel blends decrease linearly as the percentage of biodiesel in the blend increases. The percentage decrement of in calorific value of the blends B10MA00DEE00D90, B20MA00DEE00D80, B30MA00DEE00D70, B40MA00DEE00D60, B50MA00DEE00D50 and B100MA00DEE00D00 when compared to diesel is 1.1 %, 2.98 %, 4.82 %, 5.98 %, 7.82 %, 9.85 %, respectively. Conversely, the percentage increment of CV of the blended fuel over DEE and MA additives was 1.05 %, 1.2 %, 0.7 %, 1.6 %, 0.51 % and 0.73 % for the previous blends respectively. In contrast to diesel and biodiesel, DEE has a lower calorific value [23]. Unluckily, the fuel that was blended with diesel and biodiesel and contained DEE additive had a higher calorific value. This could be as a result of DEE's increased volatility, which enhances the blended fuel's burning characteristics.

#### 3.1.5 Flash and fire point

The flash point and fire point of jatropha biodiesel and its blends with diesel with and without moringa and DEE additives were conducted using Pensky Martens Apparatus according to ASTM D93 standard and the results are illustrated in Table 5. Flash point is a minimum temperature by which the fuel will ignite because of the source of ignition while the fire point is also a minimum temperature in which the fuel vapors catch flame and continue to burn once ignited, after 5 to 10 temperature differences from flash point. Both flash and fire points are used to determine whether the fuel or biodiesel is safely transportable and storable. The higher the flash and fire point of the sample, the safer for transportation and storage it will be presented by Rajak et al. [27]. The flash and fire point of jatropha biodiesel (159.6 and 167.2 °C) were higher when compared to that of diesel (52.6 and 57.3 °C) respectively, and hence it is not safer for transportation and storage. The flash point and fire point of each blends of jatropha and diesel was increasing as the concentration of the biodiesel was increasing, but the use of DEE and MA additives was decreased significantly. The lower flash point of DEE (−40 °C) caused the flash point of blends to decrease [35] and moreover it is volatile the use of 5 % DEE and 10 % MA decreased the flash and fire point of jatropha biodiesel to 107.4 and 117.2 °C respectively.

#### 3.1.6 Cloud and pour point

Table 5 illustrates the measured value of the cloud point (CP) and pour point (PP) of diesel blends and jatropha biodiesel with and without DEE and MA additives using a Simple Compression Refrigeration circuit (ET 101) in accordance with ASTM D2500 and ASTM D97, respectively. The sample's cold flow qualities are determined by the lowest temperatures, CP and PP, which also determine the sample's flow-losing features and crystal formation, respectively. A fuel is better suited for use in colder temperatures when its CP and PP are lower [36]. As presented in Table 5, the average CP and PP of diesel were −11.3 and −13.7 °C while that of jatropha biodiesel were −6.1 and −8.2 °C respectively. The CP and PP of blends of jatropha biodiesel were increasing as the concentration of jatropha biodiesel in the blend was increasing, but the 5 % DEE and 10 % MA additives were decreased those properties of blend. Hence DEE is having lower CP and PP values, it caused the jatropha biodiesel with diesel blends to decrease with the 5 % additive [26].

### 3.2 Engine performance testing

The last task was to evaluate the performance and emissions of various blends of jatropha biodiesel and diesel with and without DEE and MA additives. This was done following the experimental investigations for the production of oil and biodiesel, as well as the characterization of the physicochemical properties of jatropha and moringa oleifera oils and their respective biodiesels. The tested biodiesel blended fuel includes B00MA00DEE00D100, B10MA00DEE00D90, B10MA10DEE05D75, B20MA00DEE00D80, B20MA10DEE05D65, B30MA00DEE00D70, B30MA10DEE05D55, B40MA00DEE00D60, B40MA10DEE05D45, B50MA00DEE00D50, and B50MA10DEE05D35. Also, effects of using of 10 % (by volume) MA and 5 % (by volume) DEE additives for the improvement of physicochemical properties of the blends, performance and emission characteristics was tested on single cylinder, 4-stroke diesel engine and the results are discussed. the brake specific fuel consumptions and brake thermal efficiency of the engine were calculated using the equations: [37].

(5) $$\text{Brake}\;\text{specific}\;\text{fuel}\;\text{consumption}\;\left(\text{BSFC}\right)\;\left(\text{kg}/\text{kWh}\right)=\frac{\text{Fuel}\;\text{Consumed}}{\text{Power}\;\text{Output}\;}*3600$$

(6) $$\begin{array}{c} \text{BTE}=\text{Brake}\;\text{thermal}\;\text{efficiency}\;\left(\%\right)=\frac{Brake\;Power\;\left(kW\right)}{mass\;flow\;rate\;\left(\dot{m}\right)*\text{alorific}\;\text{value}\;\text{of}\;\text{the}\;\text{fuel}\;\left(\text{kJ}/\text{kg}\right)\left(CV\right)}\;*100\% \end{array}$$

#### 3.2.1 Brake power (BP)

**Fig. 4:**
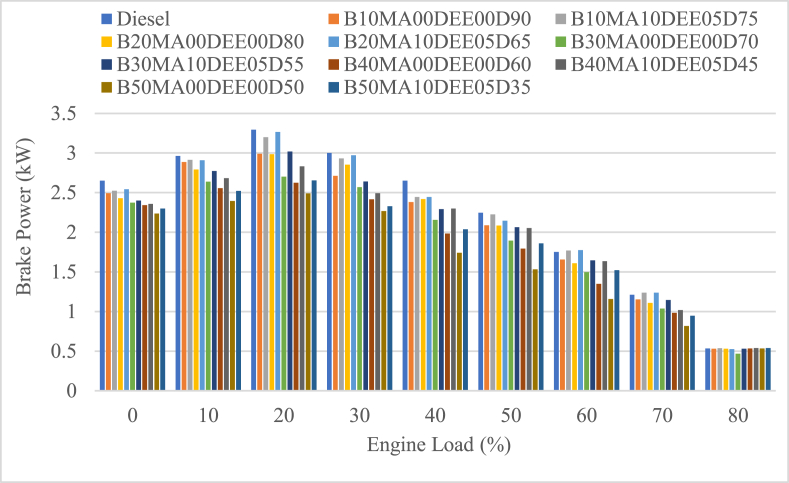
Variations of brake power with engine load.

The brake power of an engine tested with B10MA00DEE00D90, B20MA00DEE00D80, B30MA00DEE00D70, B40MA00DEE00D60, and B50MA00DEE00D50 with and without DEE and MA additive on the corresponding blends including pure diesel fuel was tested and as a function of engine load at different engine loads and is presented in Fig. 4. The average BP for B10MA00DEE00D90, B20MA00DEE00D80, B30MA00DEE00D70, B40MA00DEE00D60 and B50MA00DEE00D50 blends was 2.099 kW, 2.091 kW, 1.927 kW, 1.843 kW and 1.686 kW and the corresponding blends with 5 % DEE and MA additives was 2.199 kW, 2.203 kW, 2.057 kW, 1.991 kW and 1.856 kW respectively. When compared to the BP of diesel, which is 2.256 kW, the BP of jatropha and diesel blends without additives was decreasing as increasing the concentration of jatropha in the blend and the parental decrement for the corresponding blended fuels mentioned was 6.94 %, 7.33 %, 14.6 %, 18.34 % and 25.29 % respectively. But the use of additives improved the BP with average percentage increment of 4.72 %, 5.35 %, 6.76 %, 8.04 % and 10.13 % for B10MA10DEE05D75, B20MA10DEE05D65, B30MA10DEE05D55, B40MA10DEE05D45, and B50MA10DEE05D35 blends respectively. This shows that the power decrement of blends without additives was higher, and the used additives played a vital role in improving the BP at higher blend concentration of jatropha due to the higher cetane number of DEE (>120) improved the combustion of the blends and performance characteristics of the engine [26,28].

#### 3.2.2 Brake torque (BT)

**Fig. 5:**
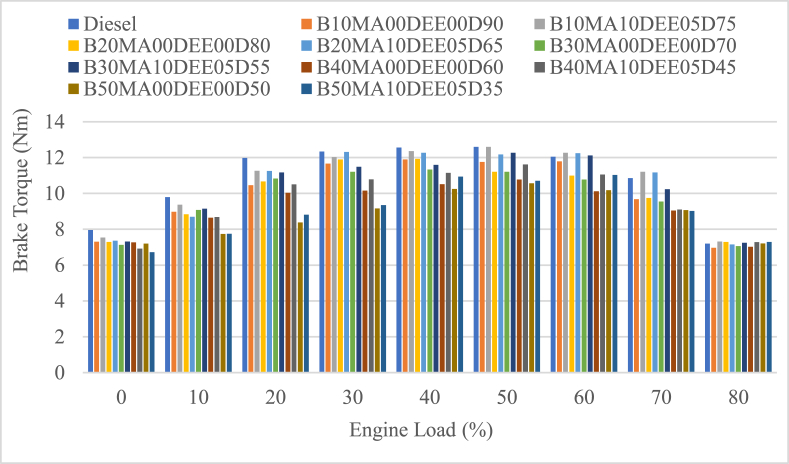
Variations of brake torque with engine load.

The brake torque (BT) of the engine run with different blends of diesel and jatropha biodiesel with and without DEE and MA additives was measured at different engine loads is shown in Fig. 5. The average BT of the engine for B10MA00DEE00D90, B20MA00DEE00D80, B30MA00DEE00D70, B40MA00DEE00D60, and B50MA00DEE00D50 was 10.053 Nm, 9.987 Nm, 9.797 Mn, 9.288 Nm and 8.860 Nm and the corresponding blends with 5 % DEE and 10 % MA additives was 10.662 Nm, 10.516 Nm, 10.286 Nm, 9.678 Nm and 9.066 Nm respectively. This implies that the average BT of the engine was decreasing when compared to pure diesel (its average BT is 10.816 Nm) as the concentration of the jatropha biodiesel in the blend was increasing. The percentage decrement for blends B10MA00DEE00D90, B20MA00DEE00D80, B30MA00DEE00D70, B40MA00DEE00D60, and B50MA00DEE00D50 was 7.05 %, 7.67 %, 9.42 %, 14.12 % and 18.08 % respectively. However, at all load conditions the use of 5 % DEE and 10 % MA played a significant role in increasing the brake torque with the percentage increment of 6.05 %, 5.3 %, 4.99 %, 4.19 %, and 2.32 % for B10MA10DEE05D75, B20MA10DEE05D65, B30MA10DEE05D55, B40MA10DEE05D45, and B50MA10DEE05D35 blends respectively. At all engine load, the blends of jatropha biodiesel and diesel with 5 % DEE and 10 % MA additives showed higher brake torque results when compared to the corresponding blends without additives and this is because of the higher volatility and oxygenated nature of DEE that cause enhanced combustion and higher turning energy of the shaft of the engine [26].

#### 3.2.3 Brake specific fuel consumption (BSFC)

**Fig. 6:**
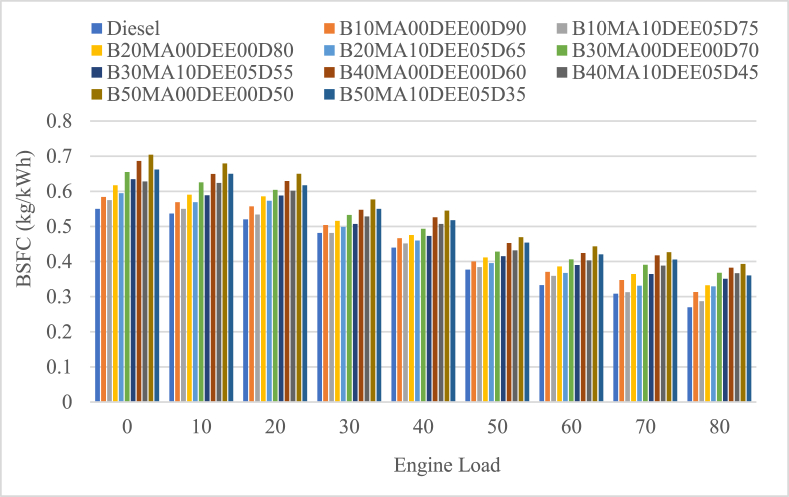
Variations of brake specific fuel consumption with engine load.

The brake specific fuel consumption (BSFC) is the ratio of the fuel flow rate per unit of power output of an engine and it is a measure of how efficiently the engine is using the fuel supplied to produce work. The BSFC of the engine for different grades of jatropha biodiesel and diesel blends and DEE and MA additives with different engine loads is shown in Fig. 6. The BSFC of the blends B10MA00DEE00D90, B20MA00DEE00D80, B30MA00DEE00D70, B40MA00DEE00D60, and B50MA00DEE00D50 was 0.457 kg/kWh, 0.475 kg/kWh, 0.501 kg/kWh, 0.524 kg/kWh and 0.543 kg/kWh and the corresponding blends with 5 % DEE and 10 % MA additives was 0.438 kg/kWh, 0.458 kg/kWh, 0.479 kg/kWh, 0.498 kg/kWh and 0.515 kg/kWh respectively. At all load of the engine, the BSFC was increasing at all the blending ratios as the concentration of jatropha biodiesel increased when compared to diesel (its BSFC was 0.424 kg/kWh), and the percentage increment of BSFC for the respective blends was 7.74 %, 12.16 % 18.04 %, 23.55 % and 28.09 %. However, the additives have significantly decreased, and the percental decrement was 4.31 %, 3.77 %, 4.27 %, 5.0 % and 5.14 % for B10MA10DEE05D75, B20MA10DEE05D65, B30MA10DEE05D55, B40MA10DEE05D45, and B50MA10DEE05D35 blends respectively. This is because of that the additives DEE and MA have decreased the density and viscosity of the blends and in turn improved the spray characteristics of the fuel and decreased the mass flow rate of the fuel as well [26,38,39]. Hence the calorific values of the blends were increased because of the use of the additives, the BSFC of the blended fuel with the additives has decreased.

#### 3.2.4 Brake thermal efficiency (BTE)

**Fig. 7:**
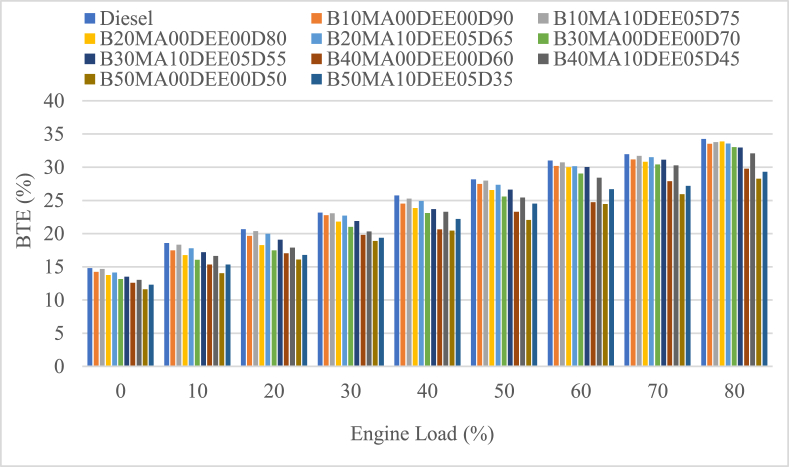
Variations of brake thermal efficiency with engine load.

Brake thermal efficiency (BTE) of jatropha biodiesel and diesel blends with and without additives was measured at different engine loads and the result is shown in Fig. 7. Based on the result, the average BTE of the blends B10MA00DEE00D90, B20MA00DEE00D80, B30MA00DEE00D70, B40MA00DEE00D60, and B50MA00DEE00D50 was 24.554 %, 23.967 %, 23.211 %, 21.232 % and 20.20 % and the corresponding blends with 5 % DEE and 10 % MA additives is 25.095 %, 24.684 %, 24.014 %, 23.04 % and 21.522 % respectively. According to the result, the BTE of the blends of JB and diesel were found to be lower when compared to pure diesel (its average BTE is 25.365 %) as concentration of the biodiesel in the blend was increasing. The percentage decrement of the mentioned blends when compared to diesel was 3.20 %, 5.51 %, 8.49 %, 16.3 % and 20.36 % respectively.

However, the use of 5 % DEE and 10 % MA additives improved the BTE with a percental increment of 2.2 %, 2.99 %, 3.46 %, 8.52 % and 6.54 % for the blends B10MA10DEE05D75, B20MA10DEE05D65, B30MA10DEE05D55, B40MA10DEE05D45, and B50MA10DEE05D35 blends respectively. The BSFC of B40MA00DEE00D60 was the maximum out of the other blends, and hence the BTE of B40MA00DEE00D60 on the contrary was found to be the minimum because BSFC and BTE are inversely proportional with each other (5), (6). The best atomization character and oxygenated nature of DEE Makes the combustion to be enhanced and the brake power to be increased; and also, the lower heat of combustion temperature cause the heat lose to be lower and the BTE to be higher [26,35].

#### 3.2.5 Exhaust gas temperature

**Fig. 8:**
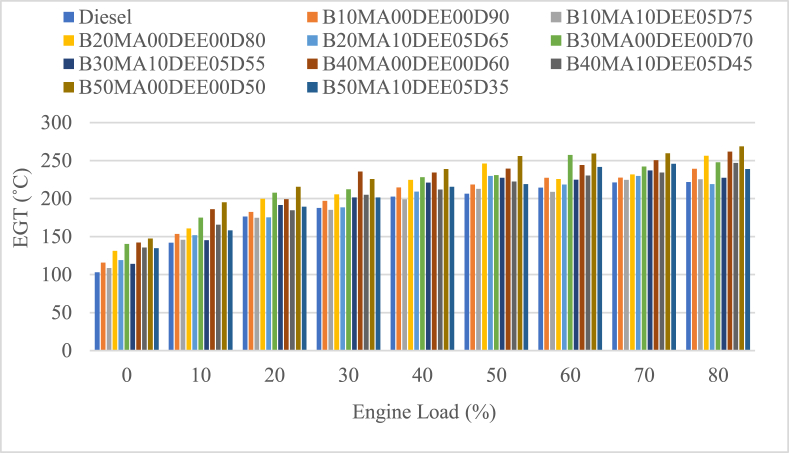
Variations of exhaust gas temperature with engine load.

The temperature of exhaust gases leaving the engine is a measure of the engine's combustion chamber temperature. The exhaust gas temperature EGT of the engine fueled with JB and diesel blends including DEE and MA additives was tested and the result is presented in Fig. 8. The average EGT of blends B10MA00DEE00D90, B20MA00DEE00D80, B30MA00DEE00D70, B40MA00DEE00D60 and B50MA00DEE00D50 was 197.3 °C, 209.1 °C, 215.8 °C, 221.5 °C and 229.6 °C while the EGT of the respective blends with 5 % DEE AND 10 % MA additives was 187.3 °C, 193.5 °C, 198.9 °C, 204.1 °C and 204.9 °C respectively. At lower engine loads (higher engine speed ranges), the EGT of the JB and diesel was increasing as the ratio of jatropha in the blend was increasing when compared to diesels' average EGT (186.2 °C). This is because of the higher viscosity and density of biodiesels' affect atomization to be poor and cause ignition delay period to be longer and the fuel to be accumulated in the combustion chamber, which in turn causes the temperature of exhaust gases to be increased when combustion starts [28,40]. Moreover, at higher engine loads (>40 %), the blends without additives depicted higher EGT values because the lower engine speed allowed the higher ignition delay period of the fuel to be attained well. However, the EGT of jatropha biodiesel and diesel blends with DEE and MA additives decreased exhaust gas temperature when compared to the respective blends without additives and pure diesel as well. The decrease in percentage of EGT with 5 % DEE and 10 % MA additives was 5.1 %, 7.5 %, 7.8 %, 7.85 % and 10.7 % for B10MA10DEE05D75, B20MA10DEE05D65, B30MA10DEE05D55, B40MA10DEE05D45, and B50MA10DEE05D35 blends respectively. The use of DEE additive causes the blend to have lower viscosity and density that also causes the improvement of atomization and combustion quality, and finally the EGT to be decreased as well [35].

### 3.3 Engine exhaust emission analysis

In this section, engine exhaust emissions that are recorded during the engine performance testing are presented. The emission test was conducted on JB and diesel blends with and without DEE and MA additives at different engine loads. The recorded resulted was interpreted and discussed in accordance with diesel and investigating the effect of DEE and MA additives with respect to the blends without additives. Exhaust emissions like carbon monoxide (CO), carbon dioxide (CO_2_), unburned hydrocarbon (HC) and nitrogen oxides (NO_x_) are presented and discussed that are recorded when testing pure diesel, B10MA00DEE00D90, B20MA00DEE00D80, B30MA00DEE00D70, B40MA00DEE00D60, and B50MA00DEE00D50 and diesel blends including 5 % DEE and 10 % MA additives on the corresponding blends.

#### 3.3.1 Carbon monoxide (CO) emissions

**Fig. 9:**
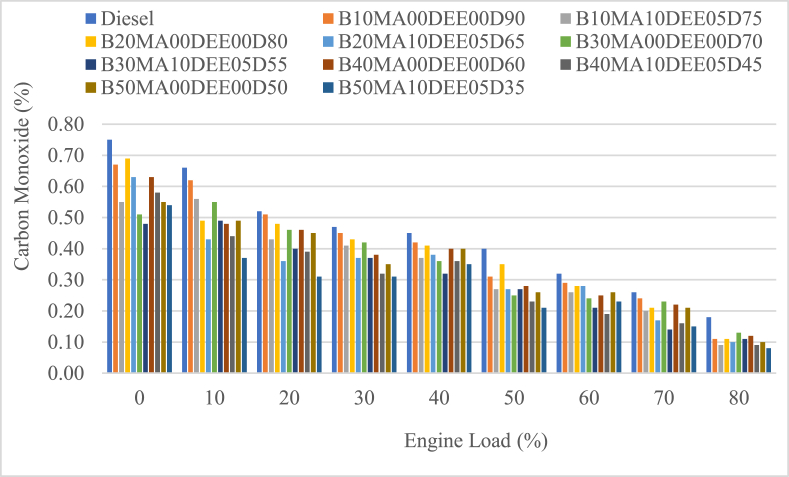
Variations of carbon monoxide with engine load.

The main cause for the formation of carbon monoxide (CO) emissions is the nature of mixture, too rich or too lean mixtures. Too lean mixtures cause the flame not to propagate all over the mixture and partial combustion cause the formation of CO and on the other hand too rich mixture cause CO formation because of insufficient oxygen available to combust the entire mixture [35]. The CO emissions of jatropha biodiesel and its blends with diesel and the effect of DEE and MA additives was on the respective blends was determined and are presented in Fig. 9. According to the result, the mean CO emissions of B10MA00DEE00D90, B20MA00DEE00D80, B30MA00DEE00D70, B40MA00DEE00D60, and B50MA00DEE00D50 blends was 0.40 %, 0.38 %, 0.35 %, 0.36 and 0.34 % while the CO emissions of the corresponding blends with 5 % DEE and 10 % MA additives was 0.35 %, 0.33 %, 0.31 %, 0.31 % and 0.28 % respectively while the and that of diesel was 0.45 %. When compared to diesel, the blends of jatropha biodiesel and diesel was decreasing as the ratio of jatropha biodiesel in the blend was increasing and the percental decrement of the blends was 9.73 %, 14.0 %, 21.5 %, 19.7 % and 23.4 % for B10MA00DEE00D90, B20MA00DEE00D80, B30MA00DEE00D70, B40MA00DEE00D60, and B50MA00DEE00D50 blends respectively. Moreover, the use of 5 % DEE and 10 % MA additives over B10MA00DEE00D90, B20MA00DEE00D80, B30MA00DEE00D70, B40MA00DEE00D60, and B50MA00DEE00D50 blends also further reduce CO emissions by 13.26 %, 13.3 %, 11.4 %, 14.3 % and 16.94 % when compared to the respective blends without additives respectively. As shown in the figure, CO emission was significantly decreased at higher loads of the engine than that of lower loads because at lower loads the speed of the engine is higher and cause the combustion process to be incomplete and CO emission increases [25,40]. However, DEE additive showed CO emission to be lower because of its oxygenated and volatile nature cause the combustion to be improved and the CO emissions to be lower [25,35].

#### 3.3.2 Carbon dioxide (CO_2_) emissions

**Fig. 10:**
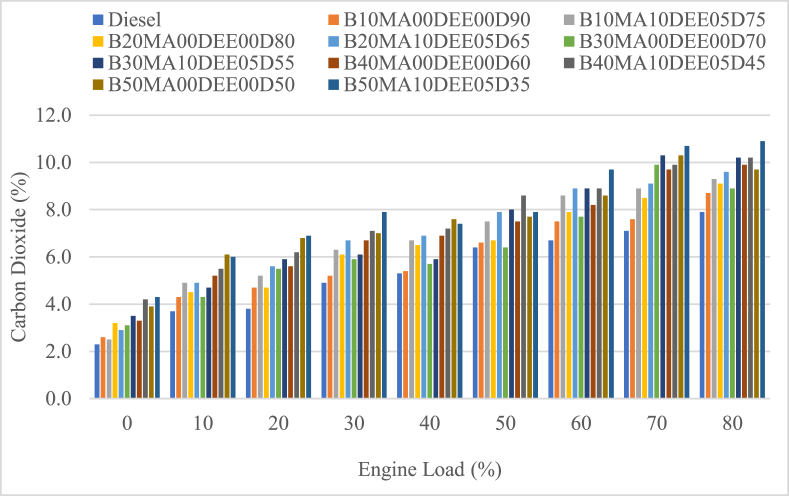
Variations of carbon dioxide with engine load.

Emissions of carbon dioxide (CO_2_) for the jatropha biodiesel with diesel blends with DEE and MA additives was analyzed at different engine loads and is presented in Fig. 10. For the entire engine loads, the emission of CO_2_ was increasing as the engine load was increasing and it shows the complete combustion of the air-fuel mixture due to sufficient delay period. The average CO_2_ emissions for B10MA00DEE00D90, B20MA00DEE00D80, B30MA00DEE00D70, B40MA00DEE00D60, and B50MA00DEE00D50 blends were 5.8 %, 6.4 %, 6.4 %, 7 % and 7.5 % while the corresponding blends with 5 % DEE and 10 % MA additives was 6.7 %, 6.9 %, 7.1 %, 7.5 % and 8 % respectively. As per the result, CO_2_ emissions of jatropha biodiesel and diesel blends was increasing as the blend ration of jatropha in the fuel was increasing and the percentage increment of B10MA00DEE00D90, B20MA00DEE00D80, B30MA00DEE00D70, B40MA00DEE00D60, and B50MA00DEE00D50 blends was 9.35 %, 18.92 %, 19.34 %, 31 %, and 40.8 % respectively, when compared to diesel (its average CO_2_ was 5.3 %). And, the percental increment of the blended fuels because of the use of 5 % DEE and 10 % MA additives was 13.9 %, 9.3 %, 10.6 %, 7.62 % and 6 % for B10MA10DEE05D75, B20MA10DEE05D65, B30MA10DEE05D55, B40MA10DEE05D45, and B50MA10DEE05D35 respectively. Because of the continual burning trend, the increased CO2 emissions for jatropha biodiesel and its blends with diesel may be a sign of complete combustion. Furthermore, compared to the corresponding blends without additives, the blended fuels with DEE additives had higher CO2 emissions due to their increased volatility and cetane number, which improved combustion [26].

#### 3.3.3 Unburned hydrocarbon (HC) emissions

**Fig. 11:**
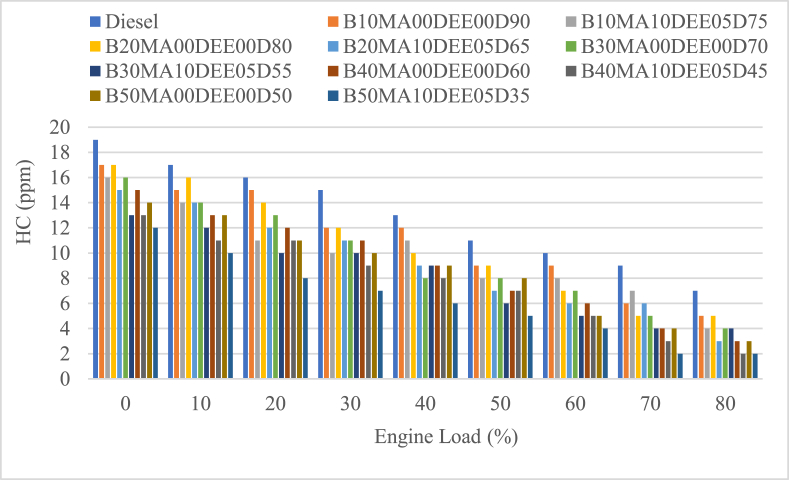
Variations of unburned hydrocarbons with engine load.

As shown in Fig. 11, unburned hydrocarbon (HC) emissions of diesel blends and jatropha biodiesel with and without DEE and MA additives were examined in relation to engine load. The mean HC value of the diesel was 13 ppm, and the corresponding blends with 5 % DEE and 10 % MA additives were 9.89 ppm, 9.22 ppm, 8.11 ppm, 7.67 ppm, and 6.22 ppm, respectively. The blends with B10MA00DEE00D90, B20MA00DEE00D80, B30MA00DEE00D70, B40MA00DEE00D60, and B50MA00DEE00D50 were 11.11 ppm, 10.55 ppm, 9.55 ppm, 8.89 ppm, and 8.56 ppm. The blended fuel's HC was falling for the whole engine load while the blend's percentage of biodiesel was rising. When comparing the blends B10MA00DEE00D90, B20MA00DEE00D80, B30MA00DEE00D70, B40MA00DEE00D60, and B50MA00DEE00D50 to diesel, the corresponding percentage decline was 14.53 %, 18.8 %, 26.5 %, 31.63 %, and 34.19 %.

Furthermore, the use of 5 % DEE and 10 % MA additives showed a slight decrease in HC emissions and the percentage decrement of HC emissions for B10MA10DEE05D75, B20MA10DEE05D65, B30MA10DEE05D55, B40MA10DEE05D45, and B50MA10DEE05D35 blends when compared with the corresponding blends without additives was 11 %, 12.6 %, 15.1 % and 13.8 and 27.3 % respectively. The reduced HC emissions of JB and its blends with diesel is because of the higher oxygen content of jatropha JB cause the combustion to be enhanced and the cylinder temperature to be increased.

This in turn causes the combustion to be completed and HC emissions to be lowered [38,41]. On the other hand, without being affected by its higher density and viscosity that cause poor atomization and incomplete combustion, MA additive resulted decreased HC emissions when compared to the corresponding blends without additives because of its higher degree of oxidative stability characteristics [34]. However, the probability of adding higher concentration of DEE additive in the diesel biodiesel blend cause the combustion temperature to be lower and causes the formation of HC emissions at the combustion chamber walls due to incomplete combustion [41]. In general, hence the concentration of DEE is lower (5 %) and that of MA is slightly higher (10 %), HC emission slightly decreased when compared to the respective blends without additives.

#### 3.3.4 Nitrogen oxides (NO_x_) emissions

**Fig. 12:**
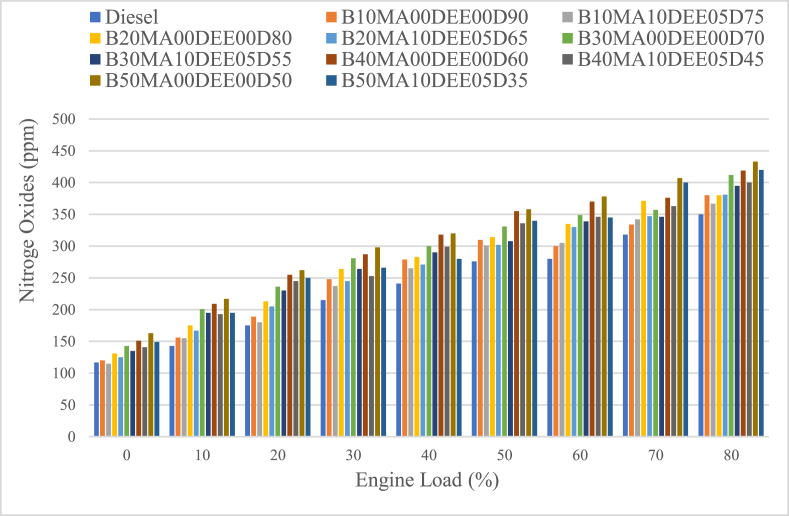
Variations of nitrogen oxides with engine load.

Emissions of nitrogen oxides (NO_x_) as a function of engine load are presented in Fig. 12. As the engine load was increasing (decreasing the engine speed), the NO_x_ emission for all blends was increasing because of the sufficient time for complete combustion to take place at increased temperature [35]. As per the result, the average NO_x_ emissions of B10MA00DEE00D90, B20MA00DEE00D80, B30MA00DEE00D70, B40MA00DEE00D60, and B50MA00DEE00D50 blends were 257.33 ppm, 274 ppm, 290 ppm, 304.4 ppm and 315.1 ppm that were increasing as the concentration of jatropha in the blend was increasing when compared to diesel by which its NO_x_ emission was 235 ppm. The percentage increment of the blends B10MA00DEE00D90, B20MA00DEE00D80, B30MA00DEE00D70, B40MA00DEE00D60, and B50MA00DEE00D50 when compared to that of pure diesel was 9.5 %, 16.6 %, 23.4 %, 29.6 % and 34.1 % respectively.

Whereas, the average NO_x_ emissions of the blends B10MA10DEE05D75, B20MA10DEE05D65, B30MA10DEE05D55, B40MA10DEE05D45, and B50MA10DEE05D35 were 251.9 ppm, 263.7 ppm, 278 ppm, 286.2 ppm and 293.9 ppm respectively. The increase in percent of NO_x_ emissions of the blended fuels B10MA00DEE00D90, B20MA00DEE00D80, B30MA00DEE00D70, B40MA00DEE00D60, and B50MA00DEE00D50, as compared to diesel, were 9.5 %, 16.6 %, 23.4 %, 29.5 %, 34.1 % respectively. However, the blended fuels B10MA10DEE05D75, B20MA10DEE05D65, B30MA10DEE05D55, B40MA10DEE05D45, and B50MA10DEE05D35 showed decreased NO_x_ emissions when compared to the corresponding blends without additives and the percentage decrements were 2.12 %, 3.8 %, 4.14 %, 6 % and 6.8 % respectively. The biodiesels' higher oxygen content available in their chemical structure made the combustion temperature to be increased and NO_x_ emission to be higher when compared to that of crude diesel [28]. But, a decrease in NO_x_ emission of the blends due to 5 % DEE and 10 % MA additives was observed because of the use of MA reduces the free radicals’ formation that cause NO_x_ to be formed during combustion of the fuel [42], and the use of DEE that is having high latent heat of vaporization over the blends also play a vital role in reducing the combustion temperature of the engine that in turn cause the formation of NO_x_ to be lower [28].

## 4 Conclusions

The effects of adding moringa oleifera biodiesel as an additive in addition to DEE were examined experimentally, and the findings on various blend ratios of blended fuel including a blend of diesel and biodiesel were presented as a potential replacements for traditional energy sources. Therefore, the following conclusions are drawn from results and discussion part.

o Compared to different biodiesel-diesel blended fuels, the addition of moringa oleifera biodiesel additives greatly enhanced the physicochemical properties, performance metrics, and emission characteristics of the engine operating on these blended fuels. Also, DEE improved the physicochemical characteristics of the biodiesel blend when it was added to Jatropha biodiesel. To assess the combined impact of the MA and DEE additions on the blended fuels, the concentrations of these additives were 10 % and 5 % by volume, respectively. Based on the findings, the final 10 % MA addition, which had an induction period of 14.22 h, demonstrated superior stability characteristics compared to the 3 %, 5 %, and 10 % MA additives over the jatropha biodiesel. DEE significantly contributed to the enhancement of the blended fuels' density, viscosity, flash point, and cold flow characteristics because of its oxygenated nature.
o In comparison with blended fuels without additives, DEE increased the engine's BSFC and BTE. Considering the tested samples, B50MA10DEE05D35 displayed a larger loss of BSFC with a percentage decrement of 5.14 % than B50MA00DEE00D50. When compared to the same blend without additives, the blended fuels with additives demonstrated higher BTE on blend B40MA00DEE00D60 with a maximum percentage rise of 8.52 %.
o In comparison to blended fuels without additives, the additives demonstrated higher CO2 emissions and decreased CO, HC, and NOx emissions in terms of emission characteristics. When compared to the biodiesel blend without additives, B50MA10DEE05D35 demonstrated the greatest reduction of CO, HC, and NOx among all the blended fuels, with percentage decrements of 23.4 %, 27.3 %, and 6.8 %, respectively.

## 5 Future work

Comparing B50MA10DEE05D35 blend against B50MA00DEE00D50, however, CO2 increased by 8 %. The large cylinder temperature during the combustion of the blended fuels causes the stable diatomic nitrogen molecule to combine with oxygen to generate nitrogen oxides, which is why NOx emissions were found to be higher for the complete fuel blends than for diesel fuel. The oxygenated nature of the compounds and the greater cylinder temperature combination result in higher CO2 emissions. Adequate oxygen is available since the elevated cylinder temperature also leads to the anticipated CO emissions splitting into CO2. Therefore, future research will focus on using other additives to reduce CO2 without affecting other combustion parameters.

## CRediT authorship contribution statement

**Tewodros Taye Birhanu:** Methodology, Investigation, Formal analysis, Data curation. **Dinku Seyoum Zeleke:** Supervision, Project administration, Conceptualization.

## Declaration of generative AI and AI-assisted technologies in the writing process

During the process of preparing this research work the author(s) did not use any form of AI or AI assisted tool or service. Therefore, the author(s) take(s) full responsibility for the content of the publication.

## Declaration of competing interest

The authors whose names are listed immediately below certify that they have NO affiliations with or involvement in any organization or entity with any financial interest (such as honoraria; educational grants; participation in speakers’ bureaus; membership, employment, consultancies, stock ownership, or other equity interest; and expert testimony or patent-licensing arrangements), or non-financial interest (such as personal or professional relationships, affiliations, knowledge or beliefs) in the subject matter or materials discussed in this manuscript.

## List of Abbreviations

ASTM: American Society for Testing and Materials
BSFC: Brake specific Fuel Consumption
BT: Engine Brake Torque
BP: Brake Power
BTE: Brake Thermal Efficiency
CI: Compression Ignition
CO: Carbon Monoxide
EN: European Standard
HC: Hydrocarbons
KV: Kinematics Viscosity
MB/A: Moringa Biodiesel/Antioxidant
NO_x_: Nitrogen Oxides
RCOOH: Carboxylic Acid
